# 563. Hydro-capillary Hydro-conductive Dressings in Burn Care: Outcomes from a Prospective Cohort Study

**DOI:** 10.1093/jbcr/irag033.303

**Published:** 2026-04-06

**Authors:** Wayne George Kleintjes, Tarryn Kay Prinsloo

**Affiliations:** Sheik Khalifa Hospital Fujairah, Cape Town, Western Cape; Cape Peninsula University of Technology, Cape Town, Western Cape

## Abstract

**Introduction:**

Burn wounds often require advanced dressings that balance exudate control, infection prevention, and an optimal moisture environment to promote healing. Hydro-capillary hydro-conductive dressings have been developed for moderate- to high-exuding wounds, combining high absorbency with a non-stick layer designed to facilitate ease of use. Despite their growing clinical application, limited evidence exists regarding their efficacy in the burn care setting of the current Low- Middle-Income Country. This study evaluated the performance of such a dressing in a tertiary burn center.

**Methods:**

A prospective descriptive observational study was conducted from September 2021 to July 2024, with a target enrolment of 70 patients. Eligible wounds included partial- or full-thickness burns with measurable exudate. The study dressing was applied beneath a low-adherent absorbent layer and covered with a porous cotton bandage. Exclusion criteria were wounds with overt infection, where bactericidal dressings were indicated. Data collected included demographics, % total body surface area (TBSA), wound characteristics, and standardized wound photographs. Wound assessment followed the BE ME *SIC* SOS framework. Pain was recorded using a visual analogue scale with pictorial “smiley face” indicators.

**Results:**

Sixty-two patients were enrolled (38 male, 24 female), including 30 donor sites. The most common injury mechanism was flame burns (n = 55), followed by hot water (n = 3), electrical burns (n = 3), and oil burns (n = 1). Mean age was 35 years, and mean TBSA was 29.7%. Across the cohort, no wound infections, odor, biofilm, or swab requirements were observed. Slough was visibly reduced, and wounds demonstrated favorable colour changes (pink to red) with progressive epithelialization. Exudate was effectively absorbed, while wounds retained sufficient moisture to promote healing. The dressing was not associated with pain. Overall, the dressing was well-tolerated and supported positive healing outcomes.

**Conclusions:**

The hydro-capillary hydro-conductive dressing demonstrated clinical effectiveness in managing exudative partial- and full-thickness burns in this cohort. The dressing supported optimal moisture balance, exudate control, slough reduction, and epithelialization, without causing pain or infection-related complications.

**Applicability of Research to Practice:**

These findings suggest that hydro-capillary hydro-conductive dressings provide a practical, patient-friendly option for burn wound management, particularly in high-burden clinical settings. Their ability to reduce slough, manage exudate, and promote healing with minimal patient discomfort may help optimize resource use and improve patient quality of care. Further comparative studies could clarify their cost-effectiveness and potential role in standard burn care protocols.

**Funding for the study:**

N/A.

